# “Obviously It's for the Victim to Decide”: Restorative Justice for Sexual and Family Violence From the Perspective of Second-Wave Anti-Rape Activists

**DOI:** 10.1177/10778012231174353

**Published:** 2023-05-11

**Authors:** Daye Gang, Maggie Kirkman, Bebe Loff

**Affiliations:** School of Public Health and Preventive Medicine, Monash University, Melbourne, Australia

**Keywords:** restorative justice, gendered violence, sexual violence, family violence, feminism

## Abstract

While the appropriateness of restorative justice for sexual and family violence continues to experience worldwide feminist debate, these programs already exist. We thematically analyzed the transcripts of former members of a second-wave feminist antirape organization in Australia to ascertain their perspectives on a victim-centered conferencing model. They provided informed and valuable insights drawn from their decades of advocacy. Participants supported restorative justice in principle, stressing respect for victims’ agency and independence in all policies and program designs. Their reservations, consistent with concerns in the literature, related to meeting victims’ needs within a constrained system rather than principled opposition to the concept.

## Introduction

While there is no consensus on the definition of restorative justice for sexual and family violence, at its core is the emphasis on the “repair of harms and of ruptured social bonds caused by crime; it focuses on the relationships between crime victims, offenders and society” (Daly & Immarigeon, 1998, p. 22). The perpetrator will have admitted responsibility, explicitly or implicitly (Daly, 2016), and the process allows parties to “resolve collectively how to deal with the aftermath of the offence and its implications for the future” (Marshall, 1996, p. 37).

Restorative justice is increasingly offered as a justice option for criminal offences and other harms in many countries. While restorative justice programs were initially developed for crimes such as assault and robbery (Wood & Suzuki, 2016), it has been available for sexual and family violence, though less frequently, for several decades (see, e.g., Couture et al., 2001). We have identified 13 restorative justice programs globally that have been the subject of empirical peer-reviewed research, including two in Australia (where the authors are based). Braithwaite (1989), an early and influential author in the field of restorative justice, described a theory of State-based reintegrative shaming for criminal offenders. In developing his theories, he drew from social practices in countries with low crime rates such as Japan. Today, restorativeness is a feature of certain measures adopted within the criminal justice system in Australia, such as victim impact statements and financial compensation offered to victims of crime (Victims of Crime, 2021). Two notable programs funded and run by the government are the South Australian youth justice system since 1994 (Courts Administration Authority of South Australia, 2012) and New South Wales since 1999 (Bolitho, 2015).

In the jurisdiction of Victoria, a community-based program was run by the South Eastern Centre Against Sexual Assault and Family Violence in Victoria (Loff et al., 2019) that was independent of the criminal justice system although the service itself is funded by government. The Victorian Department of Justice and Community Safety has recently commenced operating the Family Violence Restorative Justice Service. The victim-centered conferencing model used by both these programs is the predominant one in Victoria. It has been adopted in other restorative justice processes in Victoria for adults, including the generalist Victim-Centred Restorative Justice Program (Government of Victoria, 2022), and the Restorative Engagement and Redress Scheme for Victoria Police employees (Government of Victoria, 2021). These initiatives take place despite four decades of reforms to sex offences so the conventional criminal justice system can better suit complainants’ needs (Gang et al., 2022). As a jurisdiction, Victoria has made meaningful inroads in its efforts both to reform the conventional system and offer innovative options to victims of gendered violence.

### Debate on the Appropriateness of Restorative Justice for Gendered Violence

Reforms to the conventional justice system in many jurisdictions have not produced all the changes hoped for in the treatment of survivors of gendered violence, defined in this paper as sexual and family violence (see, e.g., Daly & Stubbs, 2006; Naylor, 2010). Survivors remain marginalized in prosecutions (Naylor, 2010, p. 663), and some are retraumatized by their participation in the prosecutorial process (Koss & Achilles, 2008, p. 3). Some feminists now argue that restorative justice for sexual and family violence may be a justice option that can be responsive to the needs of survivors, and perhaps perpetrators (see, e.g., Mills et al., 2013), in ways that cannot be accommodated within the conventional criminal justice system.

However, there is substantial controversy over the application of restorative justice processes to these crimes. Some feminist scholars fear that restorative justice for sexual and family violence will produce a shift in which adult perpetrators vacate the conventional criminal justice realm for private settings, allowing perpetrators to avoid meaningful accountability (reviewed, e.g., by Daly & Stubbs, 2006, p. 18). On the contrary, the criminal justice system risks co-opting restorative justice programs in service of the carceral and colonial state (Baldry et al., 2015; Kim, 2020), as abolitionist feminists fear has happened in the United States (Kim & Kanuha, 2022). There are doubts that a restorative justice process will prevent retraumatization if the process fails to address the power imbalance that underpins sexual and family violence (Stubbs, 2004, pp. 9–10). Furthermore, it is unlikely that an entrenched pattern of behavior will change after one restorative justice process (Daly & Stubbs, 2006, p. 17). Thus, it is argued, restorative justice would represent a move by society to a weaker response to sexual assault (Curtis-Fawley & Daly, 2005, p. 610). Other feminist scholars are favorably disposed to a restorative justice approach and are building the evidence for it (Gang et al., 2021; Marsh & Wager, 2015), but the fundamental debate on appropriateness has not been laid to rest and may never be.

To contribute to the debate, we sought the perspectives of former members of a feminist antirape activist organization who challenged the political, social, legal, and medical myths surrounding rape and sexual assault in supporting victims, while refusing co-option by government. As part of the vanguard of the Women's Liberation Movement in the 1970s and 1980s in Victoria, Australia, they offered then-novel critiques and suggestions for reform. These women have continued to develop progressive ideas in their personal, social, and professional lives since their early days as activists. Given the difficulties they faced in advocating for victims and fighting for reform, it was considered that their reflections on restorative justice would produce informed and valuable insights into whether, from a feminist perspective, restorative justice may appropriately be applied to sexual and family violence.

### The First Organization in Victoria Against Rape

Women Against Rape (WAR) was founded in Melbourne, Victoria, Australia, in November 1973. It opened the Rape Crisis Centre a year later, on November 25, 1974 (Victorian Women's Liberation and Lesbian Feminist Archives, 2009). It was the first and key organization in Victoria to combat rape and its causes. WAR provided support to women who had been raped (Taylor, 2018) in an era where no such service existed, and support could not necessarily be expected from family, friends, the police, or other services.

WAR aimed to analyze and confront rape in a feminist framework to empower and support women who had been raped (Victorian Women's Liberation and Lesbian Feminist Archives, 2009). Members operated a Rape Crisis Centre in the form of a phone information, referral, and support service; accompanied victims to police stations, hospitals, and courts; organized marches such as the Reclaim the Night Marches (Hewitt & Worth, n.d.); and wrote and published a resource booklet titled “WAR on Rape” (Women Against Rape Collective, 1977). They also participated in government processes addressing rape through legislation and policy, with a vocal presence on the government “Rape Study Committee” (Hewitt & Worth, n.d.).

Members of WAR worked to reshape social constructs around women, gender roles, and rape, based on their own reading, weekly discussions, and reflection. As part of a larger research project, we aimed to understand what former members of WAR think of restorative justice as an option for sexual and family violence in light of their past experience and current circumstances.

## Method

A qualitative method was appropriate because “experience, meaning, and perspective” were sought from participants to understand personal and social meaning (Hammarberg et al., 2016). We sought to interview all former members of WAR who consented to being interviewed, in order to capture comprehensive personal perspectives. To accommodate socially embedded personal histories that inevitably incorporate new meanings and understanding that come with decades of reflection and experience, we chose to use narrative theory as our interpretative frame. We position ourselves within Bruner's (1986, 2004) theory of the narrative mode of thought (in contrast with the rational mode of thought) in which explanatory stories link significant events and themes in ways that enable people to make sense of their lives. Bruner's insights have been used in research that investigates subjective meaning in personal accounts (Kirkman, 2002). We did not set out to identify a narrative typology but to encourage rich accounts of interpreted experience and knowledge. To encourage personal reflection and unexpected insights, we asked open questions and pursued what was important to the participants rather than to our preconceptions.

### Recruitment

All former members of WAR were eligible to be interviewed. In the absence of a comprehensive list of members, the first author contacted members who were known to the research team. These women identified or notified other former members until the population of potential participants was exhausted and invitations to participate had been distributed. In the few cases where no contact details were available, the woman's name was searched online, and messages were left on her website or Facebook page.

An invitation in which the research was briefly described was emailed to all eligible women. Those who expressed interest were sent a Participant Information and Consent Form. Volunteers could choose to be interviewed face-to-face, by telephone, or over Skype at a time of their convenience. Consent forms were signed and returned before or at the time of the interview.

The project-specific interview guide sought each woman's reflections on her experience as a member of WAR as well as her views on restorative justice. Other topics included the criminal justice system and victims’ experiences of and choices after rape. We gave a brief description of restorative justice as a process where the perpetrator and the victim of a crime come together with a convenor, and occasionally other support persons, to discuss the impact of the crime and what can be done to move on from it. We also collected information on each participant's age and occupation during WAR membership and at the time of interview, period of association with WAR, and place of residence. Each woman was asked if she wanted to use her own name or choose a pseudonym. At the end of the interview, the women were invited to communicate anything else they wanted to say by email or in a subsequent interview.

Interviews were conducted by the first author guided by at least one other author. Each interview began by asking what the participant recalled about WAR and followed the interviewees’ narratives. The interviewer encouraged participants to expand on matters that were important to them. Interviews were audio-recorded, with permission, and transcribed verbatim. Participants were reminded that they could ask for the recording to be paused or ended and that they could exclude material from analysis.

The restorative justice conferencing model discussed with participants by the researchers was a victim-centered conferencing model where the conference is designed largely to meet victims’ needs and allow the perpetrator, or a person who exacerbated the impact of the sexual assault, to take responsibility for harm. Although there are several other models of restorative justice, such as Circles of Support and Accountability (Duwe, 2013) and victim impact panels (Zosky, 2018), they largely aim to rehabilitate offenders and were not described to participants. We therefore did not make any comment about the role of the government and the place of the program, if any, within the criminal justice system, and did not first mention other alternative justice measures such as mediation or victim impact statements.

### Thematic Analysis

While maintaining our narrative approach we identified themes in each person's account and across all accounts using the six steps of a standard process (Braun & Clarke, 2006). Transcripts were read several times and validated against the audio recordings. We first sought themes inherent in topics we had defined, as listed in the interview guide, such as ideas about restorative justice, then searched for unexpected themes. As each new theme was identified, all transcripts were read to ensure that no examples were omitted. All themes and subthemes were interpreted in the context of each woman's narrative as a whole rather than as a separate entity. A hierarchy of themes was developed by testing several versions and deciding which best organized the data. This process began by grouping subthemes within an initial set of major themes. Some subthemes could fit under more than one theme heading and prompted discussion of whether they might, instead, constitute a major theme. Arrangement and rearrangement continued, with constant reference to the original accounts, until the authors agreed on a pattern that best did justice to the meaning conveyed by each participant. Illustrative quotations were then selected, ensuring that all participants were represented. The process of analysis was subject to frequent review and discussion within the research team until consensus was reached.

### Ethical Considerations and Reflexive Statement

The research was approved by the Human Research Ethics Committee of Monash University (No. 9360).

The authors are women and feminists, aged in their twenties, sixties, and seventies. The two older researchers, both white, spent most of their lives in Australia and are contemporaries of WAR members. The youngest researcher, who is Asian and subscribes to intersectional and socialist feminisms, is familiar with the cultural context of women in North Korea, South Korea, and Australia, and has been following the current feminist movement in South Korea, which reflects many of the themes of the Women's Liberation Movement. The authors took care to avoid convenient assumptions about shared feminist values, recognizing the contribution of diverse feminisms and the significance of their historical context (Burton, 1992).

All authors have been engaged in feminist thought and advocacy for most of their adult lives, and one of the authors is a former member of WAR. Both her insider experience (Probst, 2016) and expertise in restorative justice were valuable perspectives for the research. The research team was aware of her dual role. She did not take the lead in any interviews, did not participate in analysis of her own interview, and took care to acknowledge and maintain any differences of perspective between herself and other participants. Given the intention of interviewing all surviving members, it would have been inappropriate to exclude the researcher, who was already a central part of the team in previous work. The inclusion of one author as a participant did not therefore distort the results.

Rather, it was a clear advantage of this researcher's insiderness that she was able to recall the names of former members and assist with finding their contact details. However, all authors’ insider–outsider statuses became more complex upon interview, where it was revealed that WAR members had some group activities and many independent or paired activities, to the extent that there was only some overlap between participants’ experiences in WAR, including that of the author participant. As WAR was last active four decades ago, all participants had distance from their activities. The choice of method meant that this research was directed at personal rather than organizational perspectives and diverse views were encouraged. Moreover, the interviews revealed layers of insiderness to which different authors had different levels of access: One could be an insider to the feminist politics of the participants; one could be an insider to the activist experience of the 1970s and 1980s in Melbourne, Australia; one could be an insider to WAR. It is therefore difficult to say that insider experience was particularly advantageous or constitutive of bias when all participants were free to construct their own narrative of experiences long past.

Insider–outsiderness in our study therefore cannot be characterized as a dichotomy but as a three-dimensional model dependent on degree of insider- or outsiderness, or as a series of Venn diagrams the boundaries of which are fuzzy (Labaree, 2002). The team was able to balance its analysis with both outsider and insider perspectives, acknowledging that that experience was a kaleidoscopic one.

## Results

### Participants

Sixteen women were identified as active members of WAR over its lifetime. Two women had died and two declined the invitation to participate. The remaining 12 women were interviewed. Participants were aged from 60 to 74 (with a mean of 65) at the time of interviews in late 2017 and 2018. They were aged from 17 to 32 (with a mean of 22) when they joined WAR and were involved for between 1 year and 12 years (with a mean of 4.3 years). When they were involved in WAR, they were students (seven), unemployed (two), lawyers (two), and a doctor (one). At the time of interview, they were retired (four), academics (two), lawyers (two), writers (two), a counselor (one), and a consultant and director (one). All participants lived in Australia: metropolitan Melbourne, Victoria (seven); rural Victoria (one); New South Wales (three); and Queensland (one). We did not explicitly ask the race and ethnicity of the participants, but two (unrelated) participants discussed their Polish Jewish heritage and a third described her Canadian upbringing in a Hungarian refugee family.

The 12 women participated in 13 interviews: One woman required two short interviews because of other commitments, another requested a second interview to discuss archived documents, and two women requested a shared interview. Interviews took place in university offices (eight), the participants’ workplaces (two), the participants’ homes (two), or by telephone (one). Interviews took from 47 to 268 min, with a mean of 109 min. All chose to use their own name although a few requested that some details be disguised. All participants confidently gave independent and strong opinions in their interviews.

### Themes

The identified themes were *victims’ limited choices; knowledge of restorativeness;* and *relationships with government and the criminal justice system*. There were many links across themes and subthemes. Participants considered that a restorative justice process should be informed by respect for victims’ choices and would depend on victims’ character and motivations. They were aware that victims’ experience of the criminal justice system was often hostile and associated this knowledge with their concerns about how a restorative justice program might be designed. Participants were already familiar with the concept of restorativeness because they associated it with their personal and professional experiences, and because they had observed reports of restorative processes in the media. Participants were wary of how the politics of a restorative justice program might limit victims’ free choice about their participation in the program, and proposed guidance for how such a program should be established.

### Victims’ Limited Choices

Participants thought it was a positive development that some victims were now explicit about wanting to confront their perpetrator; they supported the use of restorative justice for these purposes if the victim desired it. Participants recalled that responsibility for rape was attributed to the victim rather than the perpetrator both in society and at every stage of the criminal justice system: police report, medical examination, and trial and cross-examination. That attitude was said to be reflected by many of the professionals who worked within the system. We identified two subthemes related to victims: *respecting victims’ choices*, and *victims’ experience of the criminal justice system*.

#### Respecting victims’ choices

Participants noted that it was a matter of personal importance to members of WAR to be able to seek and choose a justice option because some of those members or their friends had themselves been victimized. Participants overwhelmingly adopted the position that restorative justice should be an available option if victims wanted it. This accords with the position of WAR that victims should be supported to do as they chose in response to rape, whether they wished to report the crime or not.

Sally and Sue J. both emphasized the importance of voluntariness. Sally would “look at it from the perspective of the victim, and obviously it is for the victim to decide whether they want to participate in this or not.” Although Lyn thought restorative justice would be a means for victims to feel “validated,” she was uncertain that the rape victims who requested support from the rape crisis center would ever have wanted such an option because they felt so deeply violated: “The last thing they wanted to do was see the perpetrator, let alone talk to them. It might be a bit different if your house was burgled, but anything which is an attack on your integrity, safety, whatever: very difficult.”

Lyn did contend that many victims wanted to be heard even more than they wanted a conviction: “What did the women that we went with to trial actually feel about all that? And I think the women who actually had the opportunity to take, to go with their case and go to trial, even if the guy was found not guilty, felt that their voices had been heard. And I think that is an example of restorative justice.”

Support of victim's choices was recorded at the time in a letter to the editor written by a member of WAR that Bebe had kept. She read out the reasoning of the organization in supporting victims regardless of their choices: “One reason why rape is such a traumatic event is because the victim loses control over her own body. The feelings of helplessness that result can be overcome in part by giving her the opportunity to regain control of herself, rather than by having someone take care of her.”

Echoing the same commitment to validation, Hanna reflected that “people just often want to be heard and acknowledged.” Michelle called restorative justice a “powerful option” available to both victims and perpetrators. Meredith, referring to the victimization experienced by some members of WAR in their own lives, considered that more people would like to be able to make sense of their experience by meeting the perpetrator:

I can think of many circumstances where it isn’t, but I think … there's increasingly an understanding for many people, to be able to forgive or at least have a greater understanding of what motivated your abuser is important to the person who's been abused. For their own sense of recovery, if you like. So I think that's good that they’re doing that.

Lyn's recollection of the motivations of rape victims who called the rape crisis center was that those who:

actually had the opportunity to take, to go with their case and go to trial, even if the guy was found not guilty, felt that their voices had been heard. … That their complaint was taken seriously, they were believed, and the police were encouraged to take it further and the prosecution further, et cetera. Because just listening to what women had to say, just being believed and having a case made on their behalf, was so important that they were even prepared to go through the most horrendous cross-examination to get that heard in court. And yes, they’d be disappointed. Not just disappointed; they’d be bereft if the men were found not guilty, but it was still, for them, they felt it was better to have done it, than not have done it.

#### Victims’ experience of the criminal justice system

Participants observed that they worked within a society that attributed responsibility for rape to the victim, rather than holding the perpetrator accountable for their actions. Meredith suggested that victims of rape who called the rape crisis center for support were aware that they would not be received kindly by the criminal justice system: “People felt extremely ashamed and unable to talk too freely about what had happened to them, and did not necessarily expect that, if they did speak to someone, that they would be sympathetic or would believe them.”

Indeed, Lalita described the process of reporting a sexual assault to police as fraught with hostility and discouragement:

It was very difficult to convince police to even take a statement about an offence having been committed. That's the first thing. The second thing is, even if they believed you, they would counsel you against doing it, very strongly. And they would say, “Look, it is really a horrible procedure. You know, you have to understand what you’re getting yourself in for. You have to understand what this will involve, what it will involve in terms of taking a statement, taking physical evidence, having you examined, and then your word against this man's word, and you know, there would be a lot of pressure not to proceed.” And that was across the board for a very, very, very—and probably still now—but for a very long time, significant pressure not to do anything.

Lalita attributed this hostility to the attitudes of the police officers who were gatekeepers to the criminal justice system: “Either the police thinking that the woman was a slut, and they should not have to deal with her, or just not wanting to waste their time because nothing happened with these complaints.” Bebe told of a talk she gave about rape at a detective training school: “One of the trainee detectives said, ‘A woman cannot be raped unless she wants to be raped; it is like trying to thread a moving needle. All she has to do is cross her legs.’ And nobody disagreed.”

Meredith recalled the Police Surgeon at the time, who she said trained medical students by using his own photographs of the injuries of women he had examined, but that he may have neither obtained consent for their use nor deidentified them before use. Sue J. recalled this Police Surgeon saying that “he watched the way women took off their clothes when they were getting undressed for the examination because that told him a lot about them.”

These attitudes persisted into the courtroom. Lyn described prosecutions for rape as “schemozzles” and criticized the jury direction requiring corroboration of the victim's evidence, “even if she was so frightened that she was going to be killed, or he’d stuffed a rag in her mouth, or had his hand over her mouth. … What kind of a hue and cry can you make?” Sally recalled a practice allowed by the laws on evidence and procedure at the time: “the fact that you could have the friends and relatives and girlfriends of the accused sitting in court whilst the victims were giving evidence.” In one case, Sally heard a defense barrister joke in court that the intellectually disabled complainant of a rape case could not “keep her cunt shut.”

Sally had herself been a witness in the rape trial of a woman she knew and had been alarmed at the way the complainant's status in the courtroom was diminished to that only of a witness: “The entire tenor of the courtroom was … you don’t have standing, and as a matter of law that's right. … You don’t have a special stake in the outcome of this case. You’re just a witness.” This was not the only member who recalled defense barristers having hostile attitudes to victims and their support persons. Michelle supported a victim who had asked for assistance from the rape crisis center during her perpetrator's trial. She recalled how the criminal barristers acted aggressively toward the victim and her supporters merely because they were present: “There was no need for them to do that. They were defending their client. They could have sat with their client and done their job. So to even think that they had a right to try and intimidate either us or the young woman who was alleging the rape, was just, yeah, totally unacceptable and a real instance of male power.” She said that this behavior continued into the courtroom during the cross-examination of the victim, “questioning the type of woman she was, and that she was really there, and she consented by the very nature of what she does, she consents. … She was terribly intimidated.”

In addition to having been a juror, Lyn had given expert medical evidence in several trials for sexual assault over the years and had seen a positive change in the professionals involved in the criminal justice system, who now “come across as believing the woman. … I think a lot of [the evidence-gathering] is around making sure that they’re supporting the woman's credibility.” However, she thought it remained the case that for many victims the experience of access to justice for sexual assault has not improved significantly. Hanna, who after being active in WAR went on to work in women's refuges for a long time, observed:

There's an ongoing real problem in relation to what juries do, and how women who are sexually assaulted get treated. So, I mean, I think that that's a really interesting legal and societal dilemma ‘cause you’ve now got those instructions to the jury in terms of how they deal with certain information and make their decisions, but it's actually changed nothing, as far as I understand.

### Knowledge of Restorativeness

Many participants had personal or professional experience of processes they considered restorative in nature, or awareness of justice processes such as mediation and victim impact statement that they associated with restorative justice and restorativeness. They therefore considered restorative justice to have potential. We identified two subthemes related to knowledge of restorativeness: *experience of concepts associated with restorativeness* (incorporating the subthemes of *personal experience* and *professional experience*), and *restorative justice in existing processes*.

#### Experience of concepts associated with restorativeness

##### Personal experience

When asked which position WAR might have adopted in relation to restorative justice, Meredith replied that members would have respected victims’ right to choose because some were themselves victims or friends of victims:

There would be some people who would say that, … because of the power imbalance, it's not going to work, and others who would say that, well, people can make their own choices about that. And I think that's probably where we would have landed with restorative justice, because, again, you know, we had lived experience. And so, for some of us, it would have been really important to hear that person say, “I’m sorry,” and to give them the opportunity to say that they were sorry. Even if that doesn’t. … And also just to say to them what impact it had had on you as a person.

One of Sue J.'s close friends had been raped by the friend's father, and she remembered this friend “wanting to go and tell him what a bastard he’d been.” She therefore saw the need to support those who chose that option. Sally, who felt that she had been bullied by a member of WAR while others participated or looked on, associated the idea of restorative justice with her confrontation with the organization at a meeting:

I came back, … basically, to confront them. … Came back probably three or four months later. Not with the intention of re-joining the collective, but in order to say, “This is what you did. And this is, you must not do this to another woman. … I’m not coming back, I’m here simply to make sure that you are aware of what you’ve done so that you don’t do it again.”

She connected this experience to restorative justice, concluding that it could also be an appropriate option for survivors of sexual or family violence.

##### Professional experience

Several participants referred to their work and life experiences as restorative in philosophy or in effect. Hanna had spent a large part of her career working in women's refuges. She observed that most women she had worked with preferred not to leave their abusive partner and that they wanted the relationship but not the abuse. Given that mediation is already a familiar and compulsory requirement (although not characterized as a restorative justice practice) for complex divorce proceedings in the family courts, Hanna wondered:

Am I being naïve about thinking that there's, that it's got more potential in domestic violence? But I would still say, nonetheless, yes. Because people are forced to use the Family Court anyway for arrangements. … I think it would be fantastic. I can see lots of potential and lots of potential benefits. … It's a privilege to work in the refuge ‘cause I’ve seen change, and I’ve seen it within men. … I remember one woman whose husband, like, they were refugees too, and the husband had been a shit of a husband, a shit of a father, but then when suddenly she left with the kids, and he only had access, he actually, he didn’t do anything against her and he came to be a good father, because he came to do what you would want. He appreciated his children. … I could see a lot of merit and, yeah, possibility.

Hanna also recalled a client who chose to return to a “dangerous environment” with her violent husband because of the “practical issue” that she would lose some value in her share of the family home if he, a migrant, had to pay a large fine in the event the violence came to light.

Lalita said that she practiced law in a very practical way. She would remind her clients that what they considered to be their entitlement was “a very subjective thing. And if, you know, you have to feel that how we go about this satisfies your needs, whether you get back your $50,000 that you lent or not, you need to decide that the process is something that you’re happy with.”

Michelle had seen the successful application of restorative justice in a school for intellectually disabled children in which she worked, where power imbalances among students could be significant, resulting in “bullying and power wielding.” She described the process at her school as one that separated the perpetrator and the victim so that each could receive assistance to reflect upon the impact of the behavior on them and on the broader community.

#### Restorative justice in existing processes

Participants also identified restorative practices within existing processes and mechanisms in the criminal justice system, which they thought improved access to and the quality of justice for victims of sexual abuse. Meredith pointed out that victim impact statements, taken into consideration at the sentencing stage of a criminal prosecution, were very important to some victims. Lalita and Sophie both referred to the restorative role Judge Rosemarie Aquilina played in allowing victims and their families to read out victim impact statements in her sentencing of Larry Nassar, who perpetrated sex abuse within USA Gymnastics (Mountjoy, 2019). Sophie reflected on the power it returned to victims and their families: “That's not the same as restorative justice, but that's very powerful, I think, to be able to confront, in a way, talk to your perpetrator in saying, ‘This is how it's affected me.’”

Lyn and Sally both referred to the Royal Commission into Institutional Responses to Child Sexual Abuse established by the Australian Government in 2013, which handed down its final report in 2017. They identified the work of the Royal Commission as victim-centered restorative justice.

### Relationships With Government and the Criminal Justice System

Participants were concerned that restorative justice may not genuinely promote agency and support voluntary access for victims. Participants questioned how a restorative justice conferencing model could be designed so that it would be of value to participants, especially victims, and how it would work in practice under the realities of government management. They associated these concerns with similar debates that took place during the life of WAR in relation to medical examination after rape, describing the tension between needing longevity and professionalization and maintaining the political perspective on the role and impact of rape. We identified two subthemes related to relationships with government and the criminal justice system: *voluntary victim participation and feminist informed programming under government management* and *considerations of future model*s. The latter was composed of *value to participants* and *concerns about program design*. Participants’ responses gravitated toward the assumption that a restorative justice program, in practice, would have some kind of relationship or interaction with the criminal justice system.

#### Voluntary victim participation and feminist informed programming under government management

Several participants had observed that rape victims often did not receive support on their own terms or consistent with their own choices because of social expectations to act or react a certain way. During the life of WAR, it opposed government funding for a State-run Sexual Assault Clinic in the medical setting of the Queen Victoria Hospital. Bebe encapsulated the position: “We argued that women weren’t sick, that rape crisis centers should be welcoming environments in which women were able to be supported through the processes that they chose, whether that was reporting or not reporting.”

Lyn recalled that WAR itself was committed to “supporting women who had been raped who didn’t want to go to the police but wanted just to be checked out.” Sue J. recalled that the feminist construct of rape was at stake when working with government:

So on one hand, there was the thing of, “Well, if they take it over, it loses the kind of feminist framework that we put around it, which we see is really important to it. That just—women just having somebody nicer to take the swabs doesn’t really do the whole job, you know, if you’re not talking about a different framework for them to understand their experiencing and frame their experiencing.” So that was against co-option. On the other hand is the thing of, well, our job is a revolution. These are things that should be available to women in the community. Why shouldn’t government take them over and do them? And there's some tension between those things that I think is legitimate.

She concluded: “The state should provide these things. But we still need to provide the narrative. Doesn’t mean we have to run the services.”

Lyn was worried that “some of the things that we start off working on end up being co-opted by government, et cetera, and kind of transformed into something which is actually, ends up being worse for the people, women, but also others as well.” She wondered whether a restorative justice process could be truly voluntary when adopted by government: “I suppose the issue will again be how do women feel about? Do they feel pressured into participating in this sort of project? Do they have a perception that things might be worse for them if they say no?”

Michelle had held a view against the supposed “bureaucratization” of services by government. Her later involvement in environmental politics had caused her to reconsider the role of government funding and professionalization, particularly after reassessing the limited capacity of WAR to support women over the long term:

A lot of services need to be fully funded. … Along with that comes professionalism and you need to have the checks and balances, and things can’t, often can’t remain in a voluntary—. … It doesn’t mean you can’t have volunteers supporting, but, you know, with the scale of things, where you really want people to have much better access and follow up.

#### Considerations of future models

##### Value to participants

WAR members thought it was a positive development that some victims were now explicit about wanting to confront their perpetrator and supported the use of restorative justice for these purposes if the victim desired it. Some thought that the success of such a program would depend heavily on the participant's capacity to participate and express their needs. Lalita, a senior family lawyer, thought that a survivor would have to be “empowered enough to make a complaint” to be able to access a program run by the authorities, adding that she did not think many more people were now sufficiently confident.

Sue J. said it would be a “pragmatic approach” to take if the victim could benefit from it and be “empowered throughout the process.” Susan also expressed concern about the safety of the victim in tentatively supporting a restorative justice option. She suggested that “it requires a really strong victim who understands very clearly why she's there and that she is absolutely psychologically and emotionally up to confronting this perpetrator.” Hanna was concerned that “people aren’t always prepared to acknowledge what they did,” which would not satisfy victims.

Some participants suggested that it was likely the perpetrator might derive more benefits from restorative justice than the victim. While Susan did support restorative justice in principle, she reflected carceral feminist arguments in adding that, for her, punishment was “an important part of the restoration of justice.” She also succinctly captured the requirement that the perpetrator would have to want to improve his behavior: “Does the lightbulb want to change?”

In reflecting on recent prosecutions of prominent and powerful men in Australia for historical sexual abuse, Lyn said: “I think things are a lot more open than they used to be. I think there's now permission to actually look at people in positions of power. I think it's still very difficult, but I can’t imagine in the seventies, say, that happening at all.”

She went on to suggest that the phenomenon of victims of rape wishing to confront their perpetrator signals a change in the attitudes of, and to, victims and their empowerment since WAR was active: “I think that's a real difference, because I don’t think those women would have even come near it. … And it's good that they now are. … [I think] they would have felt totally non-believed, … maybe even by us, if they’d come; that may have been their perception.”

##### Concerns about program Design

Several of the participants questioned whether the government's chosen conferencing model would ultimately allow victims to feel validated after sexual or family violence. Sue I. thought that the crimes included would have to be “less violent” or “less harmful” for such a program to work. Her view on restorative justice was that she could not determinatively conclude that it would be an appropriate justice option, only that it was “important” for the victims to benefit from it.

Sophie was also hesitant to endorse restorative justice for sexual and family violence because its effectiveness would depend on the model and purpose. As much as she thought that “in some situations that may be very cathartic and lead to a change of behavior,” she thought most people would be put at risk of retraumatization by this choice of alternative justice process. She concluded that her first preference would not necessarily be restorative justice, but did express support for survivors who chose to use it:

I would suspect that there would be sort of one in ten people who, like—look, I don’t know: I really need to eyeball him or I need to get this off my chest’ or whatever. And then that would be so terrific for her, but for quite a lot of other people it's so complex and tangled up that you would really have to think about, you know, what are we trying to achieve here, I think. So yeah. I wouldn’t immediately jump to, of all of the great things one could do, that restorative justice is the model that I would jump to.

Hanna thought that a restorative justice option could be a practical solution for those experiencing intimate partner violence who could not rely on conventional options, as long as they considered the clients’ circumstances: “Whatever the program is, it has to be well thought through. And I’m sure that whatever you would propose would consider that. But yeah, I think I do understand why people just want that acknowledgement. I fully understand that, actually.”

Sue J. warned that the process could develop into a “nice middle-class sense about how the world should work and how we should be with each other” which required “civilized conversations between survivors and their bloody tormenters. … If she wants to just yell at the bastard, give her a chance to yell at him if that's what she needs.” Jenny and Sue J. touched on the potential for transformation within restorative justice by suggesting that “reeducation sessions” for perpetrators, as in the case of domestic violence, would support a justice approach that would affirm victims’ agency. Sue J. was concerned that such restrictions on a program would only reinforce the power that a perpetrator sought in committing the crime by causing the victim to acknowledge that the crime had affected them. In that light, Sue J. wondered whether the idea proposed was indeed one of “justice” or more a question of “truth.” She suggested concepts such as “reconciliation,” “recognition,” or “responsibility” to distance the practice proposed from the construct of “justice,” which for her was closely related to law and its structures.

Hanna and Michelle touched on the same fundamental question of identity when they asked how a restorative justice program would work when the perpetrator could potentially self-incriminate in the eyes of the law. Neither participant, however, considered this fatal to the potential of such a program to benefit survivors.

## Discussion

We report the results of one of the first empirical studies on feminist activists’ reflections on the appropriateness of restorative justice for victims of sexual and family violence (see e.g., Curtis-Fawley & Daly, 2005). Our findings contribute to the global debate by drawing on the knowledge and experience of pioneers of modern Western feminist thought who forged their views during the early days of the Australian Women's Liberation Movement and who remained politically active thereafter.

Participants emphasized the importance of respecting victims’ choices, considering their experiences of a hostile criminal justice system. Their reflections on restorative justice drew on professional and personal experiences as well as observations of victim-centered justice processes reported in the media. Participants were concerned about how a conferencing model would be designed to support victims’ agency and what features such a model should or should not have in order to be of value to participants, especially victims.

All participants referred to the single focal point of “what does the victim want?” Their activities in WAR were premised on the fact that a woman should be supported in whatever choice she made within a constrained system, while recognizing that victims’ choices after rape were limited by social factors such as institutionalized victim-blaming and shame. Addressing this victim-blaming had clearly remained a priority for participants. Unsurprisingly, those participants who had continued to observe the criminal justice system from a feminist perspective remained concerned that most complainants of sexual assault did not achieve the outcomes they desired within that system, no matter how many reforms have been made to it since the days of WAR. On the other hand, participants recalled that some complainants whom WAR assisted did wish to give evidence in a trial because they wished to be heard and believed, risking the outcome of an acquittal, raising the question whether an alternative justice option can provide the vindication without the many negative experiences that accompany a criminal prosecution beyond a victim's control. The criminal justice system and restorative justice are, as Daly et al. (2013) found, probably not as antithetical in outcomes as they may initially seem: depending on the jurisdiction and the goals of participants, criminal justice may be restorative for victims and restorative justice can involve punitive outcomes for perpetrators. Outcomes are also influenced by the design of the criminal justice system or of the restorative justice program.

Given the structural barriers victims faced then and face now, and the systemic role they were required to play as little more than a witness to a State's prosecution of a defendant, participants appeared more open to alternative or additional solutions that would meet a victim's stated needs. This view is consistent with the literature in which it is argued that another choice independent of the criminal justice system should be available because of known difficulties with accommodating victims within the existing system, however reformed a jurisdiction may be (Naylor, 2010).

This cardinal directive from participants meant that the question on restorative justice for sexual and family violence was not “whether” but “how.” It reflects more recent literature exploring possibilities and perspectives, rather than holding a principled position for or against (see, e.g., Kasparian, 2014; Miller et al., 2020).

WAR aimed to challenge deep-rooted social assumptions about gender and gendered violence. It is therefore not surprising that former members quickly revealed considered opinions about restorative justice for sexual and family violence with little prompting. In no case did they advocate for the summary abandonment of the criminal justice system or imply that a given justice option or conferencing model would be suitable for victims in every case. Their criticisms and concerns were rooted in their commitment to the principle that victims should get what they need to move on from sexual assault. They asked questions such as: Who of the victim or perpetrator gets the greater benefit from restorative justice? Does the perpetrator escape meaningful accountability? Is there a risk that victims will feel coerced or may be left unsupported, or feel unsupported, if they make a choice seen as unfavorable? Is the perpetrator committed to repairing harm rather than causing further trauma? In other words, participants expressed concerns that covered the field of reasons militating against the use of restorative justice in this setting (see, e.g., Curtis-Fawley & Daly, 2005; Daly & Stubbs, 2006). Future research could use these concerns as lines of inquiry, perhaps comparing the criminal justice system and restorative justice, in the way the different branches of the South Australian youth justice system have been compared (see, e.g., Daly et al., 2013).

It is a limitation of this study that only one version of restorative justice was presented: that of a victim-centered conferencing model where the conference is designed largely to meet victims’ needs and allow the perpetrator, or a person who exacerbated the impact of the sexual assault, to take responsibility for harm, without referring to its interaction(s) with the criminal justice system. Despite this, participants approached the conferencing model with a range of philosophies and feminisms, such as carceral feminism, transformative justice, and class analysis.

Participants' perspectives recalled the many valuable perspectives in the literature about whom restorative justice is meant to benefit, and whether the victim is vulnerable to being marginalized or retraumatized because the focus of the process is not on the repair of harm to the victim (Curtis-Fawley & Daly, 2005). To this could be added the concern that criminal justice reform programs can be co-opted in service of the colonial state, such as to maintain prisons as marginally more tolerable environments, instead of supporting Indigenous self-determination (Baldry et al., 2015). The empirical research reports transformative experiences that some participants have while participating in restorative justice programs, including both victim participants and perpetrator participants. For example, in Coker (1999), the use of traditional Navajo stories that embody gender-egalitarian themes prompted perpetrators and their families to reconsider how they saw the perpetrator's family violence. Pelikan (2010) found in Austria that, of victim participants who reported no further violence from their perpetrator following victim-offender mediation, 80% contended that the mediation had contributed to this effect. Forty per cent of victim participants whose relationship with the perpetrator continued and who had experienced no further violence reported that their partner had changed as a result of the program participation. Aspects of transformative justice are also visible where a restorative justice conference is held independently and before a criminal prosecution is finalized: Westmarland et al. (2018, p. 33) reported a voluntary conference which resulted in the perpetrator avoiding a term of imprisonment because the conference allowed the victim a longer protection order and the perpetrator the chance to make reparations by successfully winding up the joint business between the victim and perpetrator. Consistency among participant responses, theory, and empirical research indicates that the transformative potential of participation in a restorative justice program merits further exploration.

It was clear that participants had continued to engage with feminist and broadly leftist ideas, deploying them easily to assess the potential of restorative justice and comparing them to contemporary justice innovations they considered restorative for the victim. These innovations, such as victim impact statements and Royal Commissions taking statements from victims, are recent, considering the amount of time that feminists and other victims’ advocates have been calling for them. Having now encountered such examples of justice that prioritize addressing victims’ needs, participants were likely to refer to them first in their responses to draw parallels to the potential of restorative justice. It is possible that existing victim-focused justice options have now made a suite of others more socially acceptable in the #metoo era. The international examples participants cited in their interviews suggest that justice innovation can be inspirational regardless of the legal or social context. Ehret (2020) even found that women with experience of intimate partner violence preferred social and community responses to intimate partner violence outside of the legal system, such as supportive friends and community denunciation. As Herman (2005) argues, such a form of vindication goes beyond restoration.

In discussing conferencing models, participants implicitly recognized the realities of government policymaking and funding. Any policy proposal likely to be enacted must be rendered politically palatable and will be subject to resource constraints within a broad portfolio of other election promises. Policy proposals will be influenced by political parties’ estimations of what will attract votes, and policymakers are concerned to meet, or at least not to outrage, societal expectations. The Victorian Law Reform Commission (“VLRC”) issued its final report on “Improving the Justice System Response to Sexual Offences” in November 2021, attributing a Chapter, and nine recommendations, to the implementation of restorative justice. This work continues the conversation started by other Australian government reports such as the Royal Commission into Family Violence final report (2016), held by the Victorian government, and the Royal Commission into Institutional Responses to Child Sexual Abuse final report (2017), held by the Australian government. The Victorian government (2021) has committed to considering each of the VLRC's recommendations and will introduce legislative amendments to adopt an affirmative model of consent for sex offences. These government responses build on past systemic reforms to address family violence and sex offending both in the conventional criminal justice system and beyond: whether participants’ concerns about co-option were valid may soon become known. For restorative justice, at least, the question of interaction with the conventional criminal justice system is worth further consideration. For example, making judges or prosecutors the gatekeepers of participation in restorative justice may reduce its availability to those who might otherwise have chosen to participate (Koss, 2014, p.1650). On the other hand, a positive restorative justice process experience may, where appropriate, lessen the severity of the sentence a perpetrator receives. Alternatively, a complainant may wish to turn down a suggestion of restorative justice in favor of giving evidence at prosecution. Availability of or participation in a restorative justice process need not itself be a reason to withdraw a prosecution. To protect against the risk of co-option, restorative justice programs should be subject to independent evaluations to ensure they meet victims’ needs and are not captured by the criminal justice system. Further research could explore the line between interaction and co-option, and more evaluations of restorative justice programs will always be welcomed by the field.

We recognize that each criminal justice system differs in, among other things, its treatment of defendants and victims. Victoria is one jurisdiction that has made progress in recognizing victims and giving them procedural rights as participants in a legal system that does not give them standing to be parties (Victorian Law Reform Commission, 2016, p.xv). As for defendants, once found guilty of a crime, they are sentenced under the Sentencing Act (1991) (Vic) by reference to the five core sentencing principles in Section 5: punishment, rehabilitation, deterrence, community protection, and denunciation. A punitive response is therefore not the only, or even principal, sentencing outcome. In fact, in Victoria, imprisonment may be considered only as a sentence of last resort (Sentencing Act, 1991 [Vic]) and community-based orders are recognized to be punitive in their own right (*Boulton v. The Queen*, 2014), meaning that applying the sentencing principle of punishment does not require incarceration.

In Victoria at least, a substantive and positive outcome from restorative justice conference participation, demonstrably genuinely and meaningfully undertaken, may have an impact on sentence. Rehabilitation might be assessed as more likely, suggesting that a community-based order is appropriate; the accused would have shown that they do not need to be specifically deterred with a more severe sentence because they have shown accountability through their own actions. A term of imprisonment might be reduced or rendered unnecessary. Given this jurisdictional context, participation in restorative justice as a complement to sentencing in Victoria could influence the sentence in a way that restorative justice could not in other jurisdictions with more punitive goals and less victim recognition or participation.

In Victoria and other jurisdictions, the project of restorative justice will no doubt remain constrained by expectations of what perpetrators are said to deserve, how victims are expected to act, and victims’ individual material realities such as their level of financial independence, ethnicity, religion, psychological resources, access to community care and social networks, visa status, and so on. There is therefore no perfect theoretical policy setting or program design capable of overcoming engrained and persistent social constructs of sexual assault, their perpetrators, and their victims, despite the sustained advocacy of groups like WAR. Future research could therefore valuably consider and propose the best that can be done in shaping a restorative (or transformative) justice conferencing model within the constraints of election promises, government funding, and the social constructs of gender that inform them.
